# Hospital based care at home; study protocol for a mixed epidemiological and randomized controlled trial

**DOI:** 10.1186/s13063-019-3185-y

**Published:** 2019-01-24

**Authors:** Ingebjørg Irgens, Jana M. Hoff, Hilde Sørli, Hanne Haugland, Johan K. Stanghelle, Tiina Rekand

**Affiliations:** 1 Sunnaas Rehabilitation Hospital, Bjørnemyrveien 11, 1450 Nesoddtangen, Norway; 2 Institute of Clinical Medicine, University of Oslo, PO Box 1171, Blindern, 0318 Oslo, Norway; 3 Department of Neurology, Haukeland University Hospital, Jonas Lies vei 71, 5053 Bergen, Norway; 4 Department of Spinal Cord Injury, Haukeland University Hospital, Jonas Lies vei 71, 5053 Bergen, Norway; 5 Sahlgrenska Academy, Institute of Neuroscience and Physiology, University of Gothenburg, Box 100, 405 30 Gothenburg, Sweden

**Keywords:** Spinal cord injury, Tetraplegia, Paraplegia, Pressure ulcer, Pressure wounds, Videoconferencing, Telemedicine, TeleSCI, Service innovation, Interdisciplinary collaboration

## Abstract

**Background:**

Individuals with spinal cord injuries (SCI) are prone to pressure ulcers (PUs) because of the loss of sensorimotor function involved as well as increased skin moisture. Treatment of PU after SCI is complicated, involving different specialties and with need for long-lasting follow-up. This study should identify risk factors for PU after SCI, and find an effective and less time-consuming treatment for the condition among different available methods for follow-up.

**Method/design:**

The first part of this research project aims to investigate the prevalence of PU among persons with SCI based on an epidemiological design. The study will identify possible risk factors for acquiring PU. A questionnaire focusing on previous and present PUs will be sent to persons who suffered SCIs between January 2004 and January 2014. In the second part we will compare two different treatment regimens of PU through a randomized controlled pilot trial (RCT) where we will compare outpatient SCI follow-up in a hospital versus outpatient follow-up from the patient’s home, using telemedicine (teleSCI) interventions. We will compare the healing of the PU in the two groups (usual care versus teleSCI). The Tissue, Infection, Moisture Edge (TIME) registration form, the Photographic Wound Assessment Tool (PWAT) and the change in the ulcer size will be used to monitor the healing. Changes in health-related quality of life (HRQoL) and the need for assistance will be assessed using the Five Dimensions European Quality of Life scale (EQ-5D), the generic Medical Outcomes Study 12-item Short Form Health Survey (SF-12) modified version, the International Spinal Cord Injury Quality of Life Data set (ISCI-QoL Data set), and the Spinal Cord Independence Measure scale, version III (SCIM III). In addition to primary outcome measures, a cost-benefit evaluation and an assessment of patient satisfaction and participation will be performed, using customized questionnaires.

**Discussion:**

The first part of the research project will reveal the epidemiology of PU after SCI, and explore the risk factors. This part enables further prevention of PU after SCI and this information will be used in the follow-up RCT. Videoconferencing in the outpatient follow-up of persons with SCI and PU will change clinical routines and facilitate interdisciplinary collaboration, communication and competence exchange among participants of the health care services. Our research protocol allows comparing methods for interaction between medical specialists at hospitals, local caregivers in the community, next of kin, and persons with SCI and PU. The RCT should identify advantages as well as challenges in the management of PU in different follow-up settings. This study aims to identify risk factors for PU after SCI, and find an effective and less time consuming treatment for the condition among different available methods for follow- up.

**Trial registration:**

1. www.ClinicalTrials.gov, ID: NCT02800915, last update 9 October 2017.
2. The National Regional Ethical Committee (REC) 2014/ 684/ REK-Nord. https://helseforskning.etikkom.no/prosjekterirek/prosjektregister/prosjekt?p_document_id=469163&p_parent_id=473640&_ikbLanguageCode=n
3. https://app.cristin.no/projects/show.jsf?id=545284
4. https://www.sunnaas.no/kliniske-studier/bruk-av-telemedisin-som-virkemiddel-til-samhandling-i-poliklinisk-oppfolging-av-pasienter-med-ryggmargsskade-og-trykksar

**Electronic supplementary material:**

The online version of this article (10.1186/s13063-019-3185-y) contains supplementary material, which is available to authorized users.

## Background

In 2016, 126 persons in Norway were admitted to hospital for specialized rehabilitation after a newly acquired spinal cord injury (SCI) [1]. SCI has a huge impact on the individual, the family, and the health care system. People suffering from SCI are at particular risk of developing pressure ulcers (PUs) due to paralysis, reduced skin sensitivity, and skin exposure to moisture for extended periods of time [2]. Travelling long distances can worsen the condition or even cause new ulcers to develop [3–6]. In addition to direct treatment-related costs, PUs also have a significant impact on patient morbidity, mortality, and quality of life (QoL) [3, 7–9]. The actual number of persons with SCIs and PUs in Norway is unknown. We currently do not know the reasons that individuals develop PUs, and the best follow-up in those with SCI who develop a PU.

A narrative review concerning the use of telemedicine in the group of persons with SCI (teleSCI) for the last 20 years demonstrated many benefits, such as a high level of satisfaction, environmental favorability, and positive health-related cost-beneficial outcomes [10]. A previous feasibility study evaluated telemedicine as a possible alternative method for follow-up in the group of persons with SCI and PU [11]. Cost-benefit analysis of this feasibility study indicated that teleSCI provides savings for public health services, and that using telemedicine in other patient groups with similar problems could be beneficial [11]. This is in line with other studies demonstrating that telemedicine will reduce costs [12–14].

### Objectives and aims

This research project consists of an epidemiological analysis, called study 1, and a randomized controlled pilot trial (RCT), called study 2. In study 1, the aim is to analyze the epidemiology of persons with SCI and past or present PUs; such an approach helps to specify the extent of this complication. An important part of study 1 will be to identify risk factors for developing PUs after SCI.

The RCT aims to investigate clinical and economic aspects of follow-up by teleSCI compared to other methods. This part will assess the interaction between the specialized health care service, the users, and the caregivers using different approaches to follow-up. An important part of study 2 will be to identify challenges and advantages using different interdisciplinary health care services.

The protocol of study 2 includes questionnaires focusing on past and present PUs, risk factors for PUs and treatment applied. The practical part aims to measure the PU’s healing, evaluation of the quality of the service offered, user participation and satisfaction, as well as logistic and socioeconomic measurements. The study adds additional information regarding epidemiology and clinical courses of PUs.

The results of study 2 will have implications on, and will potentially influence future guidelines for, services innovated, interdisciplinary outpatient treatment of patients with PUs or other time-consuming follow-up conditions.

### Hypotheses

- Epidemiological knowledge of persons with SCI and PUs will provide insight of this complication
- Compared to other methods, PU treatment and healing will be more optimal by using teleSCI for follow-up at home measured in terms of healing, user interaction, and satisfaction

TeleSCI allows specialized hospital-based interdisciplinary teams to offer safe and effective treatment at home for persons with SCI and PU

- teleSCI enables specialized interdisciplinary health care workers to interact with, and empower, the primary health care workers
- Outpatient follow-up via teleSCI will shorten the time used for medical consultations when assessing PU
- Outpatient treatment via teleSCI will save money and time, compared with traditional treatment in hospital

## Methods/design

This research project consists of study 1 and study 2. The design of study 1 is a national multicenter trial, where epidemiological data of SCIs and PUs in Norway from 1 January 2004 to 1 January 2014 will be analyzed. A prevalence analysis of PUs will be performed. A statistical analysis will reveal possible risk factors for PUs after SCI.

**Fig. 1:**
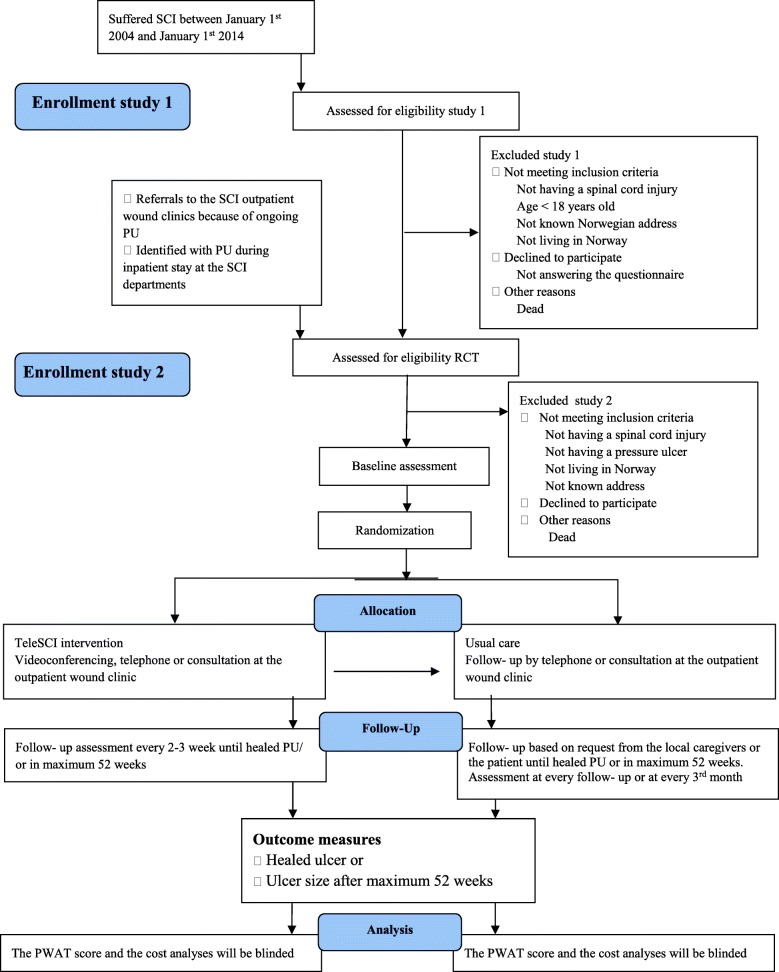
The flow diagram of the trial

**Fig. 2:**
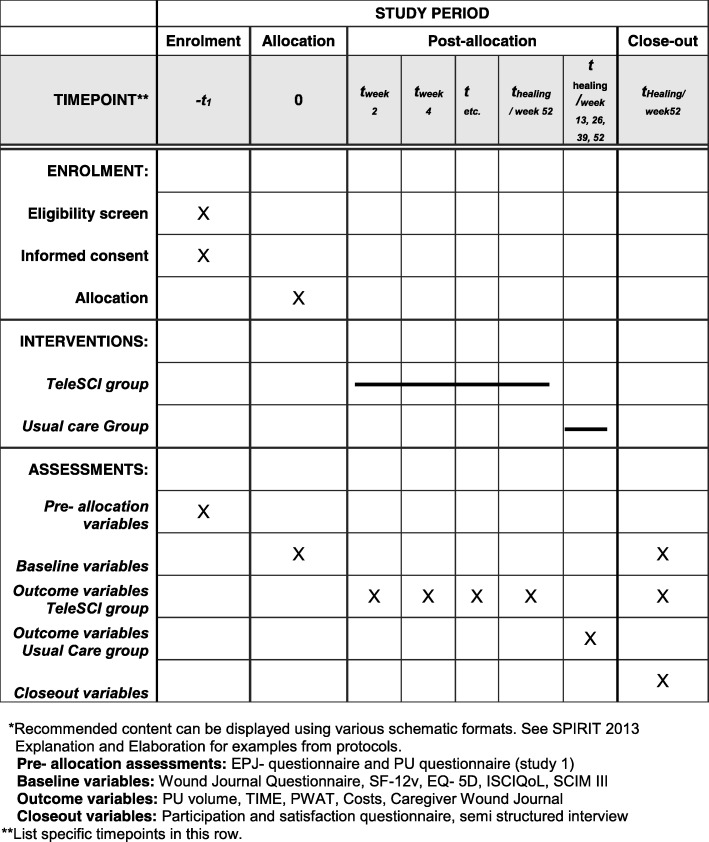
Standard Protocol Items: Recommended Items for Interventional Trials (SPIRIT). Template of content for the schedule of enrollment, interventions, and assessments in study 2.*

Individuals with SCI and present PUs will be invited to participate in study 2, which is a randomized controlled pilot trial (RCT). The participants will be randomized into two groups, where one group and their local caregivers will be offered regular, interdisciplinary, outpatient follow-up by a wound team via videoconferencing, outpatient appointments at the hospital and telephone (the teleSCI group). The other group and their local caregivers will receive guidance based on existing routines, by telephone or by outpatient appointments at the hospital (the usual care group). The only difference between the follow-up in the two groups will be that the teleSCI group will be followed by regular outpatient videoconferencing consultations from the patient’s home. The interdisciplinary wound team at the SCI departments consists of a physician, a wound nurse and an occupational therapist. A plastic surgeon and an orthopedic surgeon will be supporting when needed. The local caregivers consist of local home-care nurses, and next of kin. Both groups of patients will be followed until healing of the PU, or for a maximum of 12 months (Figs. 1 and 2). A systematic and holistic evaluation of the patient will be done at baseline. A custom made journal will be used at baseline and during the follow-up period to evaluate the treatment applied. QoL and functioning will be mapped at baseline and at the end of the follow-up period. The wound size will be measured at every assessment, using validated forms and scales. Technical logistics and costs of the health care services delivered will be mapped and evaluated using custom-made forms at every assessment. In addition, a user satisfaction and participation form will be registered either when the ulcer is healed, or after a maximum of 12 months’ follow-up (Table 1). The results from the two groups will be compared with focus on ulcer healing, QoL, user participation, and satisfaction and cost-benefit. A selection of participants in the RCT study will participate in a semi-structured interview about their experiences in the follow-up (Fig. 2 and Table 1).

**Table 1:** Measurement tools in studies 1 and 2

What	When	Study tool	Frequency
Study I	2017–18	Study 1 Form (EPJ)^a^	Once
		Study 2 Form (participant questionnaire)	Once
Study II	2015–19	“Calculating cost data”	Each consultation
		Interdisciplinary Wound Journal	Each consultation
		TIME^b^	Each consultation
		PWAT^c^	Each consultation
		SF-12v^d^	Baseline and 12 months/conclusion
		EQ-5D^e^	Baseline and 12 months/conclusion
		ISCI-QoL Data set^f^	Baseline and 12 months/conclusion
		SCIM III^g^	Baseline and 12 months/conclusion
	2018–2019	Observations, semi-structured interviews	Once

^a^*EPJ* electronic patient journal, ^b^*TIME* Time Infection Moisture Edge assessment scale, ^c^*PWAT* Photographic Wound Assessment Tool scale, ^d^*SF-12v* The 12-item Short Form Survey, ^e^*EQ-5D* Five Dimensions European Quality of Life scale, ^f^ISCI-QoL Data set The International Spinal Cord Injury Quality of Life Data set, ^g^*SCIM III* The Spinal Cord Injury Measurement scale, version III

The RCT part of the protocol conforms to the Consolidated Standards of Reporting Trials (CONSORT) guidelines extensions for randomized pilot and feasibility trials [15]. The study design is shown in the flow diagram in Fig. 1. The timeline for study enrollment, intervention, and assessment is described in the Standard Protocol Items: Recommendations for Interventional Trials (SPIRIT) [16] in Fig. 2. A SPIRIT Checklist [16] is included as Additional file 1. A summary of the questionnaires and forms is shown in Table 1.

### Ethical approval

The research project will be carried out in accordance with current ethical guidelines for health services in Norway [17], based on the Code of Ethics of the World Medical Association (Declaration of Helsinki) [18] for experiments involving humans. The research project was approved by the Norwegian Regional Ethical Committee (REC) on 9 January 2015 (2014/684 REK Nord), and registered at ClinicalTrials.gov in May 2016 (NCT02800915). The privacy rights will be followed throughout the study. Communication takes place through the Norwegian Health Network (Norsk Helsenett) using encrypted software (Cisco Meeting). The investigators will obtain informed consent from all participants before inclusion. The videoconferencing connection will be initiated from the departments offering specialized SCI rehabilitation and must be approved by the patient before log on. All videoconferencing takes place in real time and neither sound nor images are recorded or archived. The teleSCI project meets the standards and rules set by the Norwegian Data Protection Authority [19]. PUs often occurs near intimate body areas, and these areas may be visible on the screen for the participants in the intervention group. This was an ethical issue that was especially emphasized in the feasibility study [11]. The knowledge and expertise from the pilot project will be continued in the current study. Researchers from the “Distributed Home Care Solutions project” [20] also participated in the feasibility project. This ensured continuous assessment of ethical issues and user interaction throughout the project. Experience and knowledge from this cooperation has been implemented in the current research project. The Norwegian standard for information security in the health sector will be followed [19].

### Participants

#### Study 1

The epidemiological study includes all persons hospitalized at the three specialized SCI departments in Norway for primary rehabilitation from 1 January 2004 to 1 January 2014. All relevant persons identified will receive a questionnaire focusing on past and present PUs (Table 1). The study will run from January 2017 to May 2018 (Fig. 1).

#### Study 2

Sixty persons with SCIs and ongoing PUs, as well as their relatives and local caregivers, will be included in a RCT (Fig. 1). The participants will be randomized into two groups with 30 persons in each group. One group, their relatives and local caregivers will be offered regular, interdisciplinary, outpatient follow-up by the SCI department’s wound team at Haukeland University Hospital and at Sunnaas Rehabilitation Hospital via videoconferencing, telephone, and consultations at the outpatient wound clinic at the hospital (the teleSCI group). The other group, their relatives and local caregivers will receive guidance by the SCI department’s wound team at Haukeland University Hospital or Sunnaas Rehabilitation Hospital, based on existing routine. The follow-up will be offered by telephone and visits at the outpatient wound clinic at the hospital, based on request from the local caregivers or the patient. (the usual care group). The RCT will run from June 2015 to July 2019 (Fig. 1).

### Procedures

#### Identification

The participants in study 1 will be identified during an epidemiological multicentre study where the aim is to find the cumulative incidence of SCIs in Norway for the years 2004–2014, as well as to register the prevalence of PUs in this group. The time period for study 1 is January 2017 to May 2018, and the settings are all three specialized SCI departments in Norway. These three SCI departments have a life-long follow-up responsibility for all persons with SCIs living in Norway; the participants will, therefore, be recruited from municipalities from all over the country. Individuals with a SCI and present PUs found in study 1 will be invited to participate in study 2. In addition, the participants in study 2 will also be identified from patients with SCIs, currently hospitalized or referred to the outpatient clinics at the specialized SCI departments because of PUs (Fig. 1). All potential participants will be invited to be enrolled in study 2. The follow-up period will be until ulcer healing or after maximum 12 months from baseline. The settings of the RCT will be the specialized SCI department at Haukeland University Hospital and Sunnaas Rehabilitation Hospital, as well as municipalities from all over Norway (Table 2).

**Table 2:** The intervention illustrated by main features from the Template for Intervention Description and Replication (TIDieR) Checklist and Guide

*Brief name*: outpatient treatment of pressure ulcer from home by teleSCI	
*Why*: to improve the outpatient follow-up in persons with spinal cord injury (SCI) and pressure ulcer	
*What*: outpatient pressure ulcer follow-up from the participant’s home, using videoconferencing as a tool to cooperate, compared to usual care. The therapy will be tailored from the specialized health care system to the participant’s home in cooperation with the local caregivers, with focus on wound healing, quality of life, cooperation, user participation, and costs	
*Who provided*: the SCI wound team, consisting of a physician, a wound nurse, and an occupational therapist sited at Haukeland University Hospital and Sunnaas Rehabilitation Hospital. A plastic surgeon and an Ortopedic physician will be included in the wound team when necessary. The local caregivers will receive training via videoconferencing in how to treat and prevent pressure ulcer among the group of persons with spinal cord injury within the context of a clinical trial	
*How*: using videoconferencing and remote control software to a laptop at the patient’s location	
*Where*: from Haukeland University Hospital and Sunnaas Rehabilitation Hospital to the patient’s home	
*When/how much*: the experimental intervention consists of pressure ulcer treatment guidance every second or third week until the pressure ulcer has healed, or a maximum of 52 weeks.	

### Drop outs

Participation is voluntary, and withdrawal is possible at any time. The participants will still be allowed to complete follow-up treatment for their PU through the specialized health care service. Participants who for various reasons need to be hospitalized during follow-up, e.g., if needed treatment cannot be provided by local care services, or if surgery is needed, will be excluded from the study during their period of hospitalization. Length of admission and cause will be registered and the participants will be included in the study again after being dismissed from the hospital.

### Feasibility

The investigators conducted a feasibility study to explore the PU management by teleSCI before the current research project [11]. Wound healing, satisfaction, cost-benefit, and feasibility were mapped through logs, questionnaires, and semi-structured interviews with seven participants and their local caregivers. The results were promising, indicating that teleSCI is an acceptable and safe way to offer follow-up with regards to the technical solutions. Validated forms mapping QoL and function were used [21–23], as well as custom-made forms to map costs and participant satisfaction. Based on feed-back from the participants in the feasibility study [11], the forms have been improved to suit the current project.

### Screening for eligibility, baseline assessment, and recruitment

The epidemiological information concerning all relevant participants will be collected from the electronic patient journal (EPJ) system at the three SCI departments by the main investigator. The *World Health Organization (WHO) International Classification of Diseases (ICD)* [24] will be used to identify SCI-related conditions (Additional file 2). All current persons identified will receive a questionnaire focusing on past and present PUs.

### Study 1

#### Inclusion criteria

Adults (> 18 years) with incurred traumatic or non-traumatic SCI between 1 January 2004 and 1 January 2014, and who give their consent to participate.

#### Exclusion criteria

Persons with no SCI:

- Persons who are unable to give their consent due to cognitive problems
- Persons who do not have a permanent/known Norwegian address
- Age < 18 years

### Study 2

In study 2, persons with SCIs and ongoing PUs will be assessed for eligibility in a RCT. Eligible persons will be provided with detailed written and verbal information about the project, and asked to sign informed consent before inclusion. The Informed Consent Form is approved by the National Regional Ethical Committee [17]. Relatives and the local caregivers will also be informed and invited to cooperate in the follow-up study. Once the consent is given, baseline data will be collected, randomization will be done and the group allocation will be informed to the participants, either face to face during the outpatient consultation at the SCI departments, or by telephone if the participant is at home.

#### Inclusion criteria

- Traumatic or non-traumatic SCI
- Ongoing PU
- Age > 18 years
- Consent to participate given

#### Exclusion criteria

- No SCI
- No PU
- Age < 18 years
- Not living in Norway
- No known Norwegian address
- Participants who are unable to give their consent due to cognitive problems

### Randomization and allocation concealment

Participants in the RCT will be randomized directly after baseline assessment to group 1 (the teleSCI group) or to group 2 (the usual care group). The randomization will be stratified with respect to the hospitals, blocked with varying sizes, and carried out by use of the random-number generator in the statistical software SPSS. The randomization sequence list will be created in advance of recruitment, by an experienced scientist who is not a member of the research team. The randomization numbers will be provided in closed and sealed envelopes, unavailable to those who enroll the participants. At least two persons, either investigators or other medical staff members, will be present to secure the allocation procedure. If the participant is included in the teleSCI group, further arrangements for the installation of the telerehabilitation software and training in the use of the software program and equipment will be addressed to the participant, the relatives, and the local caregivers.

### Blinding

In study 1, the participants will be assigned a serial number identification within each SCI unit. This serial number will be used in the analyzing of the questionnaires. No personal identification will be used. In study 2, it will not be possible to blind the participants. The wound assessment tool [24] and the cost-benefit form will, however, be considered by blinded medical professionals not participating in the research project. Only the randomized number, not the identity of the participants, will be known during the analyzing part of study 2.

### Data collection and storage

Patients are assigned a randomly selected project identification/serial number within each SCI department in Norway. This serial number follows the individual patient through the rest of the project. The serial number and person identification form the key code. The key code and the data set will be stored, locked in different fireproof cabinets, where only the main investigator has access. Data and Telecommunications Authority’s requirements for safe information flow will be followed [25]. Only the main investigator will have access to the final trial data set. This is in line with the ethical approval of the study, given by the National Regional Ethical Committee (NREC) in Norway [17]. The main investigator will terminate the trial in 2019, and the collected results will be terminated in 2024, according to the NREC approval.

### Outcome measures

#### Study 1

Study 1 will highlight the national prevalence of PUs in persons who suffered SCIs between January 2004 and January 2014.The study will also analyze causes and risk factors of developing PUs, according to the answers in the participant questionnaires.

#### Study 2

##### Primary endpoints

Study 2 will focus on the effect of using teleSCI compared with other outpatient approaches in the follow-up of persons with PUs after a SCI, measured in terms of percentage wound size healed, and the time to heal. A systematic evaluation of the ulcer will be done, using the validated Time Infection Moisture Edge (TIME) form [26] and the Photographic Wound Assessment Tool (PWAT) scale [27], where the amount of fibrin and granulation tissue present, percentage of epithelialization of the wound, percentage of moisture present, and the appearance of the wound edges will be described. Time to heal will be measured counting days until healing, or measuring the amount of healing at the end of the 12-month follow-up period. Changes in self-reported health-related quality of life (HRQoL) and the need for assistance will be compared using the 12-item Short Form Health Survey (SF-12v) [28], the Five Dimensions European Quality of Life scale (EQ-5D) [23], and the International Spinal Cord Injury Quality of Life questionnaire (ISCI-QoL) basic data set [29]. The SF-12v [28] measures functional health and well-being from the patient’s point of view. The EQ-5D scale [23] measures mobility, self-care, activities, pain, discomfort, and anxiety/depression. The participants perform a self-rating of their health in a box-scale and on a vertical visual analog scale. In the ISCI-QoL questionnaire [29], the participants will rate their physical and psychological health, using a box-scale. The SF-12v [28], EQ-5D [23], and ISCI-QoL [29] will be used as a quantitative measure of the health outcome that reflects the participant’s own health judgment before and, at the end of, the follow-up period. The Spinal Cord Independence Measure, version III (SCIM III) scale [24] has been designed specifically for persons with SCI and is a comprehensive ability-grading scale, focusing on the ability of performing basic tasks, and the effect the SCI has on the overall medical condition and comfort. SCIM III [24] will be rated at baseline and at the end of the follow-up period, and the results from both groups in the RCT will be compared with focus on changes in performing ability during the follow-up period.

##### Secondary endpoints

The health care services as well as patient satisfaction and participation in both groups will be measured and evaluated using customized questionnaires.

In the cost-benefit form we will ask for time used at each consultation, travel distance to drive to and from the outpatient clinic at the SCI departments, as well as time used to travel, and distance driven for the local caregivers to and from the participants’ home. The number of caregivers participating in each session will be recorded, together with the total number of consultations for each participant, including teleSCI consultations, outpatient appointments at the hospital and telephone calls. Loss of working time for the participant and for any caregivers will be recorded, and so will medication and bandages used in every treatment session, both at the hospital and at home. CO_2_ emissions and costs for the environment will be mapped at every session.

Any technical problems will be mapped at every session. A custom-made questionnaire about the user’s experience with the follow-up will be mapped at the end of the follow-up period. This form contains questions about sufficient information provided before the inclusion, and during the follow-up period, as well as the participants’ experience regarding the follow-up. The results from the two groups will be compared with focus on differences in the user participation and satisfaction.

The investigators at the SCI departments at Haukeland University Hospital and Sunnaas Rehabilitation Hospital will supervise the filling in of the questionnaires and forms. After the conclusion of the follow-up period, a sample of participants will be requested to participate in a semi-structured qualitative interview based on the participants’ observation and experience.

### Adverse events

Adverse events or other unintended effects of trial interventions or trial conduct in the RCT will be collected, assessed, and reported in accordance with Norwegian legislation [30]. These possible events and effects will be reported in the trial outcomes. If the PU does not heal during the follow-up period, the participant will be invited to further follow-up also after the termination of the RCT.

### Intervention

In our previous feasibility study [11] one of the important factors regarding PU healing was that the follow-up was performed by a few dedicated homecare nurses, as this continuity resulted in a better overall overview of the follow-up. The feasibility study also showed the importance in using a simple, common description of the PU status and the plan for the further treatment. These principles will be continued for both groups in the RCT.

The location and grade of the PU, and comorbidities influencing the healing process, will lead to individualized follow-up for each participant. In case of adverse effects, or worsening of the PU, necessary therapeutic measures will be initiated to prevent further worsening of the condition. If needed, surgical treatment or inpatient care will be recommended. All adverse events and worsening of the condition will be recorded in the wound journal and in the report form. In case of bacterial infection of the PU, laboratory tests will be performed, and the participant and the general practitioner will be informed. The local caregivers will be informed with permission from the participant.

An interdisciplinary wound journal has been drafted to map current age, marital status, socioeconomic status, lifestyle factors, nutritional information, and additional conditions that may affect ulcer healing. The wound journal also contains a follow-up part where the PU and recommended treatment measures, including cushions, mattresses, pressure relief, and positioning, are continuously registered at every wound session. The wound journal was used during the feasibility study, and necessary changes were done, based on feed-back from the interdisciplinary wound team at the hospitals, and the participating patients. The questionnaire forms and time lapse of the RCT are shown in Table 1.

### The teleSCI group

The participants with SCI and PU (*n* = 30) and their local caregivers will be offered interdisciplinary outpatient follow-up every second or third week, provided by the specialized SCI department’s wound team via videoconferencing. The follow-up will consist of teleSCI appointments, visits to the SCI outpatient wound clinic at the hospital and telephone consultations. The Template for Intervention Description and Replication (TIDieR) [31] Checklist and Guide (Table 2) has been used to record and describe the intervention by teleSCI. This will secure treatment fidelity, replication for future trials, and transparency of reporting the results.

### The usual care group

The participants with SCIs and PUs (*n* = 30) and their local caregivers will receive guidance based on existing routines provided by the specialized SCI department’s wound team. Telephone contact and appointments at the SCI outpatient wound clinic will be based on initiative taken by the local health care service/patient/next of kin. As usual, as care services vary in type and frequency, the follow-up provided for each participant will depend on the local resources available.

### Fidelity

For each participant in both groups of the RCT, an interdisciplinary wound journal with questions concerning whether treatment is in adherence with the planned evidence-based treatment guidelines will be made for each consultation. The standardized report contains questions in accordance with the planned protocol, and will be used by the research investigator to ensure the fidelity of the project. A report will be sent to the participant and their general practitioner after each consultation. All forms have been created before the start of the study.

### Technical solutions [32]

In this project, we use the term “teleSCI” about computer-based videoconferencing with remote webcam and the use of telephones [33–35].

The technical solution is in line with the “Sunnaas model of telerehabilitation” [36, 37], with has also been used in other research projects concerning telerehabilitation at Sunnaas Rehabilitation Hospital [11, 32]. Based on the experiences from these projects, videoconferencing guidelines have been established at the hospital [33–35].

We will use Cisco Meeting/Acano videoconference software, and videoconferencing equipment installed at the outpatient clinic at the SCI departments at Haukeland University Hospital and at Sunnaas Rehabilitation Hospital. Participants will be given a webcam for the duration of the project. The camera will be connected to either the participant’s laptop or to a project laptop, containing downloaded, encrypted videoconferencing software (Cisco Meeting/Acano) delivered by the Norwegian Health Net (NHN). This is a company owned by the Ministry of Health and Care services in Norway. The encrypted software meets requirements with regards to data safety aspects, privacy, and confidentiality [19]. As the therapy sessions are live, there are no recordings or storage of video, sound or picture. The project wound nurse and a technician will be available to support and assist the participants should technical problems arise.

Our current technical setup builds upon experience from the feasibility study [11]. To maintain safety aspects, a checklist has been implemented to be used at the start of each therapy session to control and adjust the patient’s physical environment [34]. This is to ensure optimal follow-up conditions, preserve accurate procedures in case of emergency and to accommodate privacy issues. Technical errors will be logged in each consultation.

### Sample size

For the demonstration of effect between the two groups, sample size calculation is based on an expectation of a standardized difference of at least 0.8 (typically considered a large effect). With 80% power, we will need 25 participants in each of the two groups hence we include 30 in each group to tolerate some drop outs.

### Statistical analysis

Data from the end of the study will be analyzed using chi-square tests for categorical data and *t* tests for continuous data. Regression analyses (linear and logistic) will be performed to make any necessary adjustments for baseline differences. In addition, we will also analyze developments over time in the two groups, using the mixed-models analysis.

Mean QoL scores with corresponding 95% confidence interval will be estimated in each of the two treatment groups, and the groups will then be compared using *t* tests and mixed-models analysis. A cost-benefit analysis will be carried out to assess the cost of outpatient treatment via telemedicine, compared with outpatient treatment at the hospital.

The reduction (in percentage) of PU size is calculated with corresponding 95% confidence interval for each of the two treatment groups. The groups will be compared with regard to percentage reduction in ulcer size, using *t* tests and mixed models. All quantitative statistical analyses will be performed using the SPSS and STATA statistical software package. Computer Assisted Qualitative Data Analysis (CAQDAs) will be used in the qualitative analysis.

### Missing data

Missing data will be handled by multiple imputation [38].

## Discussion

This research project should improve, simplify, and streamline health care and health-related services. In study 1, we will explore the prevalence of PUs after SCIs, and identify risk factors for developing PUs [39]. This knowledge helps to plan outpatient services for PU patients in future. The knowledge of risk factor will contribute to further development of patient journals for PU patients.

The findings in survey 1 should form the epidemiological and clinical basis for survey 2, where we will conduct a RCT comparing innovative treatment methods for follow-up. We aim to facilitate the “Patient’s Health Care Service” through the development of an innovative, user-driven service model, based on both current, as well as future needs for treatment and follow-up, and where the model is user friendly, and create value for both the health care receivers and givers as well as for the society. Innovative telemedicine services are not necessarily simple to achieve because of the diversity of users, interests, and regulations. However, it may simplify and benefit the patients as well as health care services. This project aims to assess treatment, and to explore the different aspects of communication between care receivers, primary health care providers and specialized health care services when using teleSCI. The collaboration occurs with the specialized health care providers located on one side of the screen, while the care receiver and the local caregivers are located on the other side. This makes it simple to interact using modern means of communication, which in turn simplifies the communication process, saves time, diminishes possible risky pressure on ulcer and allows greater accessibility to specialized health care providers [40, 41]. This approach has obvious advantages compared to other telemedicine studies, based on still images of the ulcer [42].

This article describes a RCT that could make an important contribution to the field of PU healing [43], patients’ QoL and opinions about the quality of interaction and knowledge sharing between the specialized health care services and those involved in the primary health care. By conducting an economic evaluation, we will be able to determine whether teleSCI has economical benefits compared to more traditional methods [11, 13, 44, 45].

We aim to explore the impact of a new method (teleSCI) compared to the old method (usual care), with focus on the quality of the health care services offered. An important part will thus be to identify experienced challenges.

Currently, our society has an increasing aging population due to better medical care. This also includes a larger number of patients surviving severe disease or injury. Increased knowledge of potentially more accurate and effective rehabilitation in regards of SCIs and PUs will benefit society. Establishing specific recommendations on the follow-up, in both the early and chronic phase of rehabilitation plus developing tools for the monitoring, will provide useful guidance for practitioners and also enhance the accuracy of the treatment for remotely living patients.

The research project should provide knowledge about predictable, comprehensive, and evidence-based ways of management and prevention of risk factors. Optimal methods of follow-up will reduce the consumption of hospital services because a larger proportion of specialized services will be provided by municipalities. Since PUs occur frequently among patients with different chronic illnesses, such as neurological diseases, cancer, hip injuries as well as among patients, who for various reasons are bedridden for prolonged periods of time [46, 47], our project will be of value for understanding development of, and risk factors for, PUs and transferring knowledge about management also to other patient groups. The health service should offer people health care of good quality. Characteristics of good quality are that services are effective, safe, and secure, involve users, are coordinated and characterized by continuity, utilize resources in a good way and are accessible and fairly distributed [48]. There is a need for greater attention to the content and the quality, and that a service of good quality puts patients and users in the center. Our study should enable a more user-oriented health care service, with focus on systematic quality improvement work, improved patient safety and fewer adverse events. Cooperation and guidance needs a common professional platform which allows the health care system to coordinate follow-up at a local level. This is also in line with the intention of the Norwegian Collaboration Reform, offering “the right treatment at the right place at the right time” [49]. The RCT is also likely to be positive in terms of environmental impact. The use of telemedicine at the patients home leads to less pollution and expenses associated with driving to outpatient clinics. In the project we will measure and calculate traveling time, CO_2_ emissions, and costs.

## Trial status

In study 1, questionnaires have been sent to all known individuals suffering SCI in the time period January 2004 till January 2014. As of today about 50% of the population has answered. Punching of data from the study is ongoing, and the epidemiological article is expected to be ready for submission during the autumn of 2018.

In study 2, the recruitment in the RCT study started in June 2015, and about 50 participants have been recruited and randomized into the trial. Nearly 40% of the included participants have finished their follow-up period. Punching of data from the RCT will start during autumn 2018. The semi-structured interview form is in progress, and will be finished during winter 2018/2019. The trial results will be published in international peer-reviewed journals, as well as presented to the participants and the public at national and international meetings and conferences.

## Additional files

Additional file 1: Consolidated Standards of Reporting Trials (CONSORT) Checklist. (DOC 121 kb)

Additional file 2: Standard Protocol Items: Recommendations for Interventional Trials (SPIRIT) Checklist. (DOCX 28 kb)

## Abbreviations

EQ-5D: Five Dimensions European Quality of Life scale
HRQoL: Health-related quality of life
Mixed models: Fixed and random effects
PU: Pressure ulcer
PWAT: The Photographic Wound Assessment Tool
RCT: Randomized controlled trial
SCI: Spinal cord injury
SCIM III: Spinal Cord Independence Measure, version III
SF-12: The Short Form (36) Health Survey
TeleSCI: Telemedicine used in the follow-up treatment for the spinal cord injury population
TIME: Tissue, Infection, Moisture balance, and Edge of the ulcer
