# Supplementary material for: Hospital based care at home; study protocol for a mixed epidemiological and randomized controlled trial
Source: Trials. 2019 Jan 24;20:77. doi: 10.1186/s13063-019-3185-y (PMC6346520; doi:10.1186/s13063-019-3185-y)
Supplement: Supplementary file 2 — Standard Protocol Items: Recommendations for Interventional Trials (SPIRIT) Checklist. (DOCX 28 kb) [file 13063_2019_3185_MOESM2_ESM.docx]

**Additional file 2.** SCI-related diagnosis used in study 1. Based on the International Statistical Classification of Diseases and Related Health Problems 10th Revision (ICD-10)-WHO Version for; 2016

| **ICD-10 Chapter** | **Disease chapter** | **Text** | **Spesific chapter and text** |
| --- | --- | --- | --- |
| **II Neoplasms** | **C70** | **Malignant neoplasm of meninges** | C70.1Spinal meninges C70.9 Meninges unspecified |
|  | **C72** | **Malignant neoplasm of spinal cord, cranial nerves and other parts of central nervous system** | C72.0 Spinal cord C72.1 Cauda equina C72.8 Overlapping lesion of brain and other parts of central nervous system C72.9 Central nervous system, unspecified |
|  | **C79** | **Secondary malignant neoplasm of other and unspecified sites** | C79.4 Secondary malignant neoplasm of other and unspecified parts of nervous system |
| **VI Diseases of the nervous system** | **G04** | **Encephalitis, myelitis and encephalomyelitis** | G04.1Tropical spastic paraplegia G04.2 Bacterial meningoencephalitis and meningomyelitis, not elsewhere classified G04.8 Other encephalitis, myelitis and encephalomyelitis, G04.9 Encephalitis, myelitis and encephalomyelitis, unspecified |
|  | **G82** | **Paraplegia and tetraplegia** | G82.0 Flaccid paraplegia G82.1Spastic paraplegia G82.2 Paraplegia, unspecified, G82.3 Flaccid tetraplegia G82.4 Spastic tetraplegia G82.5 Tetraplegia, unspecified, |
|  | **G83** | **Other paralytic syndromes** | G83.0 Diplegia of upper limbs, G83.1 Monoplegia of lower limb,  G83.2 Monoplegia of upper limb,  G83.3 Monoplegia, unspecified G83.4 Cauda equina syndrome, Incl. |
|  | **G95** | **Other diseases of spinal cord** | G95.0 Syringomyelia and syringobulbia G95.1Vascular myelopathies  G95.2 Cord compression, unspecified G95.8 Other specified diseases of spinal cord G95.9 Disease of spinal cord, unspecified |
|  | **G99** | **Other disorders of nervous system in diseases classified elsewhere** | G99.2*Myelopathy in diseases classified elsewhere G99.8*Other specified disorders of nervous system in diseases classified elsewhere |
| **XIII Diseases of the musculoskeletal system and connective tissue** | **M46** | **Other inflammatory spondylopathies** | M46.0 Spinal enthesopathy M46.1 Sacroiliitis, not elsewhere classified M46.2 Osteomyelitis of vertebra M46.3 Infection of intervertebral disc (pyogenic) M46.4 Discitis, unspecified M46.5 Other infective spondylopathies M46.8 Other specified inflammatory spondylopathies M46.9 Inflammatory spondylopathy, unspecified |
|  | **M47** | **Spondylosis spondylopathies** | M47.0†Anterior spinal and vertebral artery compression syndromes M47.1 Other spondylosis with myelopathy M47.2 Other spondylosis with radiculopathy M47.8 Other spondylosis M47.9 Spondylosis, unspecified |
|  | **M48** | **Other spondylopathies** | M48.0 Spinal stenosis M48.2 Kissing spine M48.3Traumatic spondylopathy M48.4 Fatigue fracture of vertebra M48.5 Collapsed vertebra, not elsewhere classified M48.8 Other specified spondylopathies M48.9 Spondylopathy, unspecified |
|  | **M49** | **Spondylopathies in diseases classified elsewhere** | M49.0*Tuberculosis of spine M49.1*Brucella spondylitis M49.2*Enterobacterial spondylitis M49.3*Spondylopathy in other infectious and parasitic diseases classified elsewhere M49.4*Neuropathic spondylopathy, Incl.:  Neuropathic spondylopathy in: syringomyelia and syringobulbia, tabes dorsalis M49.5*Collapsed vertebra in diseases classified elsewhere M49.8*Spondylopathy in other diseases classified elsewhere |
|  | **M50** | **Cervical disc disorders** | M50.0†Cervical disc disorder with myelopathy |
|  | **M51** | **Other intervertebral disc disorders** | M51.0†Lumbar and other intervertebral disc disorders with myelopathy |
| **XIX** **Injury, poisoning and certain other consequences of external causes** | **S12** | **Fracture of neck** | S12.0 Fracture of first cervical vertebra S12.1 Fracture of second cervical vertebra S12.2 Fracture of other specified cervical vertebra S12.7 Multiple fractures of cervical spine S12.8 Fracture of other parts of neck S12.9 Fracture of neck, part unspecified |
|  | **S13** | **Dislocation, sprain and strain of joints and ligaments at neck level** | S13.0 Traumatic rupture of cervical intervertebral disc S13.1 Dislocation of cervical vertebra S13.2 Dislocation of other and unspecified parts of neck S13.3 Multiple dislocations of neck S13.4 Sprain and strain of cervical spine S13.6 Sprain and strain of joints and ligaments of other and unspecified parts of neck |
|  | **S14** | **Injury of nerves and spinal cord at neck level** | S14.0 Concussion and oedema of cervical spinal cord S14.1 Other and unspecified injuries of cervical spinal cord S14.2 Injury of nerve root of cervical spine S14.3 Injury of brachial plexus S14.4 Injury of peripheral nerves of neck S14.6 Injury of other and unspecified nerves of neck |
|  | **S15** | **Injury of blood vessels at neck level** | S15.7 Injury of multiple blood vessels at neck level S15.8 Injury of other blood vessels at neck level S15.9 Injury of unspecified blood vessel at neck level |
|  | **S22** | **Fracture of rib(s), sternum and thoracic spine** | S22.0 Fracture of thoracic vertebra S22.1 Multiple fractures of thoracic spine  S22.8 Fracture of other parts of bony thorax S22.9 Fracture of bony thorax, part unspecified |
|  | **S23** | **Dislocation, sprain and strain of joints and ligaments of thorax** | S23.0 Traumatic rupture of thoracic intervertebral disc S23.1 Dislocation of thoracic vertebra S23.2 Dislocation of other and unspecified parts of thorax S23.3 Sprain and strain of  thoracic spine S23.5 Sprain and strain of other and unspecified parts of thorax |
|  | **S24** | **Injury of nerves and spinal cord at thorax level** | S24.0 Concussion and edema of thoracic spinal cord  S24.1 Other and unspecified injuries of thoracic spinal cord |
|  | **S25** | **Injury of blood vessels of thorax** | S25.8 Injury of other blood vessels of thorax S25.7 Injury of multiple S25.9 Injury of unspecified blood vessel of thorax |
|  | **S29** | **Other and unspecified injuries of thorax** | S29.0 Injury of muscle and tendon at thorax level S29.7 Multiple injuries of thorax S29.8 Other specified injuries of thorax S29.9 Unspecified injury of thorax |
|  | **S32** | **Fracture of lumbar vertebra and pelvis** | S32.0 Fracture of lumbar vertebra S32.7 Multiple fractures of lumbar spine and pelvis S32.8 Fracture of other and unspecified parts of lumbar spine and pelvis |
|  | **S33** | **Dislocation, sprain and strain of joints and ligaments of lumbar vertebra and pelvis** | S33.0 Traumatic rupture of lumbar intervertebral disc S33.1 Dislocation of lumbar vertebra S33.3 Dislocation of other and unspecified parts of lumbar spine and pelvis S33.5 Sprain and strain of lumbar spine S33.7 Sprain and strain of other and unspecified parts of lumbar spine and pelvis |
|  | **S34** | **Injury to nerves and spinal cord at abdominal level, lower back and pelvis** | S34.0 Concussion and edema of lumbar spinal cord S34.1 Other injury of lumbar spinal cord S34.3 Injury of cauda equina |
|  | **S35** | **Injury of blood vessels at abdominal level, lower back and pelvis** | S35.0 Injury of abdominal aorta S35.7 Injury of multiple blood vessels at abdomen, lower back and pelvis level S35.9 Injury of unspecified blood vessel at abdomen, lower back and pelvis level |
|  | **T09** | **Other injuries of vertebra and trunk, not specified** | T09.3 Injury on the spinal cord not specified part |
|  | **T91** | **Sequelae after injury of neck and trunk** | T91.3 Sequelae after spinal cord injury T91.1 Sequelae after fracture of spine T91.2 Sequelae after other fracture of thorax and pelvis T91.3 Sequelae after injury of spinal cord T91.8 Sequelae after other specified injuries of neck and trunk |
